# Targeting Selectins and Their Ligands in Cancer

**DOI:** 10.3389/fonc.2016.00093

**Published:** 2016-04-18

**Authors:** Alessandro Natoni, Matthew S. Macauley, Michael E. O’Dwyer

**Affiliations:** ^1^Biomedical Sciences, National University of Ireland Galway, Galway, Ireland; ^2^Department of Chemical Physiology, The Scripps Research Institute, La Jolla, CA, USA; ^3^School of Medicine, National University of Ireland Galway, Galway, Ireland

**Keywords:** sialyltransferase, metastasis, niche, sialic acid, glycosylation, selectin, glycomimetic, tumor

## Abstract

Aberrant glycosylation is a hallmark of cancer cells with increased evidence pointing to a role in tumor progression. In particular, aberrant sialylation of glycoproteins and glycolipids has been linked to increased immune cell evasion, drug evasion, drug resistance, tumor invasiveness, and vascular dissemination, leading to metastases. Hypersialylation of cancer cells is largely the result of overexpression of sialyltransferases (STs). Differentially, humans express twenty different STs in a tissue-specific manner, each of which catalyzes the attachment of sialic acids *via* different glycosidic linkages (α2-3, α2-6, or α2-8) to the underlying glycan chain. One important mechanism whereby overexpression of STs contributes to an enhanced metastatic phenotype is *via* the generation of selectin ligands. Selectin ligand function requires the expression of sialyl-Lewis X and its structural isomer sialyl-Lewis A, which are synthesized by the combined action of alpha α1-3-fucosyltransferases, α2-3-sialyltransferases, β1-4-galactosyltranferases, and *N*-acetyl-β-glucosaminyltransferases. The α2-3-sialyltransferases ST3Gal4 and ST3Gal6 are critical to the generation of functional E- and P-selectin ligands and overexpression of these STs have been linked to increased risk of metastatic disease in solid tumors and poor outcome in multiple myeloma. Thus, targeting selectins and their ligands as well as the enzymes involved in their generation, in particular STs, could be beneficial to many cancer patients. Potential strategies include ST inhibition and the use of selectin antagonists, such as glycomimetic drugs and antibodies. Here, we review ongoing efforts to optimize the potency and selectivity of ST inhibitors, including the potential for targeted delivery approaches, as well as evaluate the potential utility of selectin inhibitors, which are now in early clinical development.

## Introduction

It is well established that one of the most frequent changes in cancer cells is the pattern of cell surface glycosylation (1, 2). Because glycans present on the plasma membrane influence the ability of cells to interact with their surrounding microenvironment, altered glycosylation enables cancer cells to acquire specific capabilities to interact with all components of the microenvironment, such as growth factors, chemokines, extracellular matrix, and cell to cell contact. As a consequence, processes such as adhesion, mobilization, and migration are also altered in cancer (3). The glycosylation pattern of cancer cells may also change during disease progression; indeed, metastatic cancer cells display profound differences in cell surface glycosylation, compared not only to the normal cells but also to the original tumor (4–7). Changes in glycans on cancer cells often arise from a combination of mechanisms, such as overexpression or downregulation of the glycan’s protein or lipid scaffold or modifications of the metabolic pathways responsible for the generation of specific glycans (1).

**Figure 1:**
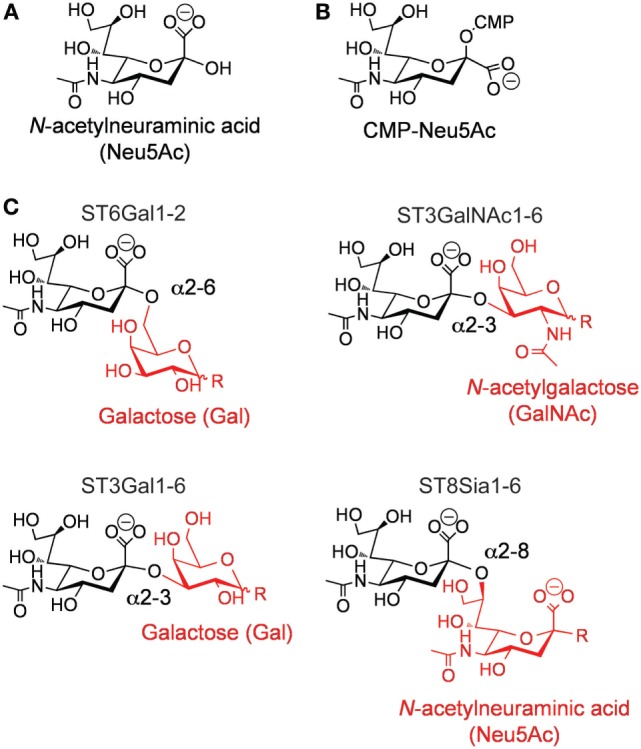
**(A)** Structure of *N*-acetylneuraminic acid. **(B)** Structure of cytidine-5′-monophospho-(CMP)-sialic acid. **(C)** Types of sialic acid linkages catalyzed by the different members of the mammalian ST family.

One of such changes in the glycome of cancer cells is the increased presence of sialic acid sugars on the surface of cancerous cells (8). Hypersialylation of tumor cells has been associated with a metastatic phenotype and inferior outcome in patients with cancer (9). Sialic acids represent a group of sugars based on the neuraminic acid scaffold, with the most frequent being *N*-acetylneuraminic acid (Neu5Ac; Figure 1A). Sialic acids are predominantly found at the non-reducing termini of *N-* and *O-*linked glycans attached to proteins or on glycolipids. Sialylation of glycans is carried out by a complex, yet highly specific, series of enzymatic processes that take place in the ER–Golgi apparatus, and are responsible for the covalent linkage of sialic acids to galactose (Gal), *N*-acetylgalactosamine (GalNAc), *N*-acetylglucosamine (GlcNAc), or to another sialic acid (polysialic acids). These enzymatic reactions are performed by a class of glycosyltranferases termed the sialyltransferases (STs).

## The Sialyltransferase Family

The ST family is part of the larger glycosyltransferase superfamily generally expressed in the secretory pathway. Recently, there has been a surge in structural understanding of the family, knowledge of which members is involved in biosynthesis of selectin ligands under both healthy and cancerous conditions, and advances in the ability to inhibit STs with a cell permeable inhibitor.

### Overview of the ST Family

In humans, the ST family is composed of 20 individual members (10–12). These enzymes are expressed in a cell- and tissue-specific manner, proving each cell type with a unique “sialome” (13). All mammalian STs use cytidine-5′-monophospho-(CMP)-sialic acid (Figure 1B) as their donor substrate and a glycan as their receptor. Exquisite stereoselectivity results in ST-catalyzed formation of an α-linked sialic acid to a precise hydroxyl group on a specific particular saccharide residue. The combination of the acceptor saccharide residue and the precise hydroxyl group on this residue can be used to divide the family into four distinct families (Figure 1C) (11, 12, 14). For example, ST6Gal enzymes catalyze the transfer of sialic acid to the 6′-hydroxyl group of a Gal residue, ST3Gal enzymes catalyze the transfer of sialic acid to the 3′-hydroxyl group of a Gal residue, ST3GalNAc enzymes catalyze the transfer of sialic acid to the 6′-hydroxyl group of a GalNAc, and ST3Sia enzymes catalyze the transfer of sialic acid to the 8′-hydroxyl group of another sialic acid residue.

All mammalian STs belong to glycosyltransferase family 29 (GT29) in the CAZy database (www.cazy.org). Only recently, the first structures of mammalian STs have been solved. The first was ST3Gal1 from porcine (15), which is the enzyme that masks the peanut agglutinin (PNA) ligand (Galβ1-3GalNAc) on complex *O*-glycans. The structure of ST3Gal1 showed for the first time how active site residues position the appropriate hydroxyl group on the acceptor for nucleophilic attack. Structures of both rat and human ST6Gal1 were also recently solved (16, 17). Fortuitously, the complex *N*-glycan on a neighboring dimeric unit was trapped in the active site of the human enzyme, providing structural insight into the well described preference of this enzyme for the α1-3 mannose branch on complex *N*-glycans. Further structural insight into this sub-family of ST awaits a cocrystal structure in which electron density of sialic acid is clearly observed. More recently, the structure of ST8Sia3 was determined, which has led to insight into how this sub-family uses a positively charged groove to position the extended polysialic acid oligosaccharide acceptor (18). Together, the structures of the first three mammalian STs provide an excellent starting point for understanding the structure basis for catalysis by the mammalian ST family. Nevertheless, there is much more work to be done in determining how different the active site architecture is between the different STs; based on differences observed in the three structures solved to date, it seems reasonable to speculate that, although challenge, it may be possible to exploit differences between members of the family to ultimately develop selective inhibitors that target individual sub-classes, or even members, of the ST family.

### Sialyltransferase Overexpression in Cancer

Many of the STs are overexpressed in various forms of cancer, which has been reviewed in detail elsewhere (19–24). In many cases, this overexpression has been correlated with cell surface overexpression of the product for that particular ST. For example, many cancer cells overexpress ST6Gal1, and as a consequence, stain strongly with the *Sambucus nigra* (SNA) lectin that recognizes sialic acid residues α2-6-linked to Gal (22). In another example, gliomas were shown to express ST3Gal1, which correlated with low staining of cells and tumors by PNA (25). On the other hand, several reports have also documented that loss or downregulation of ST expression correlates with cancer progression (26, 27). These findings highlight the need for careful studies that systematically modulate ST expression and activity to determine whether it is simply aberrant glycosylation or specifically hypersialylation that plays a role in tumor progression.

Overexpression of STs and the resultant hypersialylation in cancer has been implicated in many stages of tumorigenesis (7, 20, 21, 24). Studies have documented the roles for hypersialylation in drug and radiation resistance (28, 29). Recent work has found that hypersialylation is also involved in evasion from the immune system, with numerous types of cancer cells expressing high levels of sialylated ligands of the inhibitory receptors sialic acid-binding, immunoglobulin-like lectin-(Siglec)-7 and Siglec-9, which in turn recruit these Siglecs to inhibit natural killer (NK) cell killing (30, 31) or neutrophil activation (32). Hypersialylation is also implicated in enhancing tumor invasiveness by enhancing cellular proliferation and motility through constitutive activation of pathways involved in cell growth and motility (33, 34). A critical role for hypersialylation in cancer metastasis has also been suggested for certain types of cancer. For example, sialylated ligands of the Selectin family of adhesion proteins ligands have been described on multiple myeloma (MM) cells (35, 36) and breast cancer cells (37) and have been shown to be critical for homing and metastasis of these cancer cells. Similar observations are suggested based on correlative studies in renal cell carcinoma (38) and lung cancer (39). Based on the broad therapeutic interest around preventing cancer metastasis, this aspect is described in more detail in the following section.

## Selectin and Their Ligands in Cancer Metastasis

**Figure 2:**
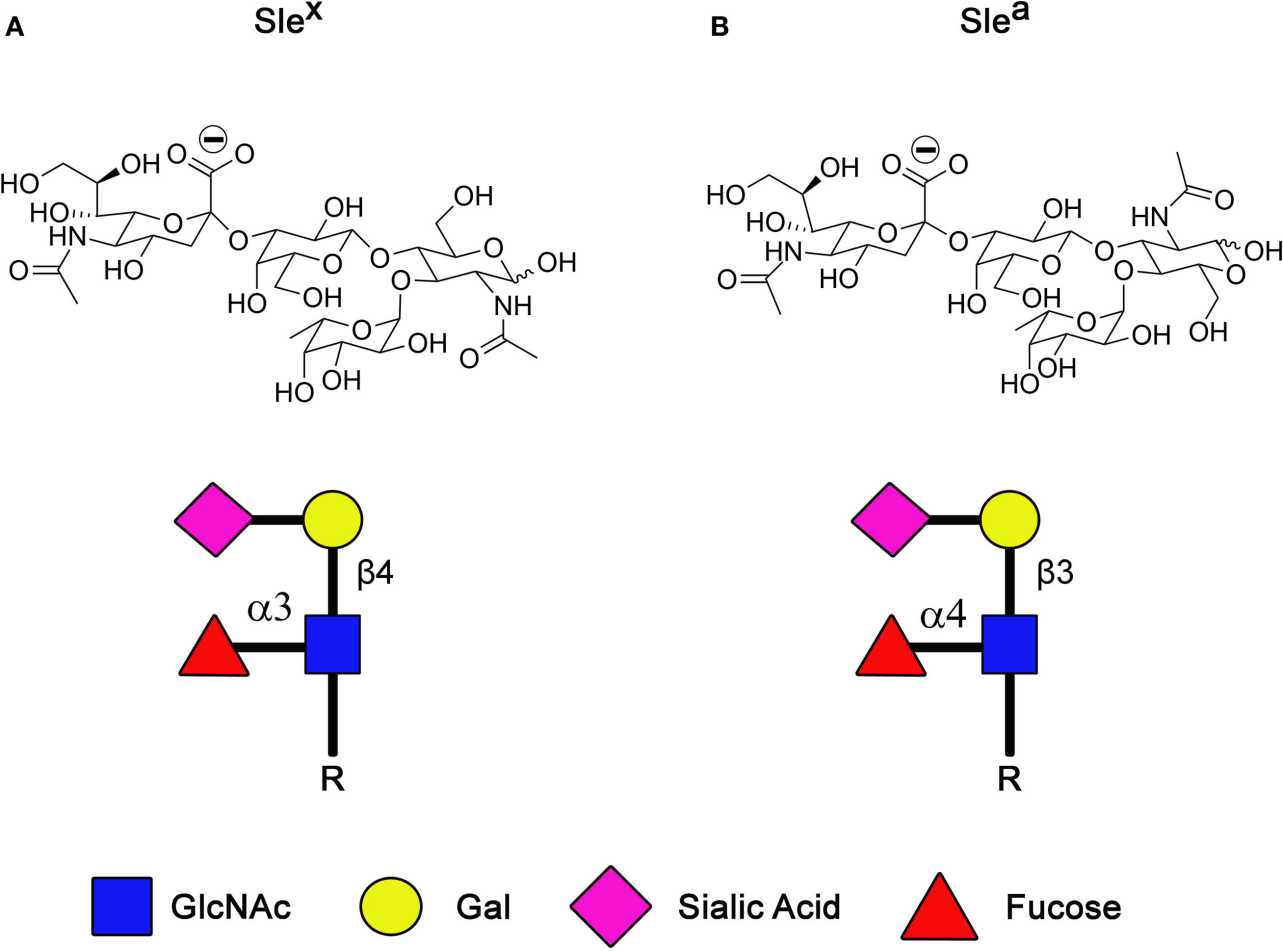
**Structure of SLe^x^ (A) and its structural isomer SLe^a^ (B)**.

Sialic acids are incorporated within many different carbohydrate structures, including sialyl Lewis X (SLe^x^) and its isomer sialyl Lewis A (SLe^a^; Figure 2). These tetrasaccharide structures are composed of α2-3-linked sialic acid on the GlcNAc backbone. SLe^x^ and SLe^a^ represent the minimal recognition motif for ligands of selectins, a family of lectins whose functions are well characterized as mediators of leukocytes trafficking (40, 41). Three types of selectins have been described so far, the L-, E-, and P-selectins. Selectins are type I membrane proteins composed of a N-terminus C-type lectin domain followed by an epidermal growth factor (EGF)-like motif, a series of consensus repeats, a transmembrane domain, and a short cytoplasmatic tail. By interacting with SLe^x^ and SLe^a^ containing glycoproteins and glycolipids, selectins are responsible for the slow tethering and rolling of leukocytes on the vascular endothelium that is the first step of leukocytes extravasation during inflammation or lymphocytes homing. As is often seen during oncogenic transformation, cancer cells take advantage of this physiological process to spread and colonize to distant organs during the metastatic cascade (3, 42). Indeed, extravasation of tumor cells during metastasis is the best documented function of selectins and their ligands in cancer (43–45). However, recent evidence suggests a role of selectins/selectin ligands interactions beyond the extravasation process, such as emboli formation, formation of a permissive microenvironment for metastasis, and retention of tumor cells in protective niches.

### Selectins and Their Ligands during Extravasation and Homing of Cancer Cells

Selectins have been directly implicated in tumor extravasation for their ability to support tumor cell rolling on activated endothelium in a process that mirrors leukocytes extravasation. In a small cell lung cancer xenograft model, it has been shown that cancer cells rolled *in vivo* on tumor necrosis factor α-(TNFα-) treated vessels, although the rolling velocity was seven times faster than that of leukocytes (46). Moreover, when cancer cells were transplanted in E- and P-selectin knockout (KO) mice, the number of metastases was significantly lower than wild-type (WT) mice, demonstrating the importance of selectins in metastases. The capability of a particular tumor to extravasate and therefore metastasize might correlate with the expression levels of selectin ligands. In colon carcinoma, there was a direct association between the levels of E-selectin ligands and the metastatic phenotype (47). Colorectal cancer cells expressing high levels of SLe^a^ extravasate more efficiently than cancer cells with low SLe^a^ *in vivo*. Similarly, the highly metastatic breast cancer cell line ZR-75-1 establishes stronger interactions with E-selectin *in vitro* compared to the less metastatic cell line MCF7 (48). Curiously, Mucin-1 (MUC-1) serves as the E-selectin ligand in these cells but also enables firm adhesion by binding to intracellular adhesion molecule-1 (ICAM-1) suggesting that selectin scaffolds may have more than one role during tumor extravasation. Mouse prostate cancer cells engineered to overexpress fucosyltransferase (FUT)3, 6, and 7, show different ability to roll on E-selectin *in vitro* with the FUT6-overexpressing cells displaying the highest number of rolling cells (49). Accordantly, the FUT6-overexpressing cells exhibit the highest metastatic potential in the bone compared to FUT3 and FUT7-overexpressing cells. Recently, we have shown that MM cells overexpress ST3Gal6 and have high levels of α2-3-linked sialic acid on the cell surface, suggesting a role of selectin ligands in homing of MM to the bone marrow (36). Knocking down ST3Gal6 decreased the levels of α2-3-linked sialic acid, impaired transendothelial migration of myeloma cell *in vitro*, and importantly reduced homing *in vivo*, indicating that the levels of selectin ligands influences myeloma trafficking into the bone marrow niche.

The ability of tumor cells to interact with selectins has been proposed to correlate to some extent with disease progression. In MM, we observed that the expression levels of ST3Gal6 increased with disease progression (36). Moreover, the P-selectin glycoprotein ligand-1 (PSGL-1) gene expression was found to increase with disease progression and, more recently, the percentage of potential E-selectin ligands bearing primary myeloma cells was shown to be higher in relapsed versus diagnosed patients (50, 51). Similarly, in acute myeloid leukemia (AML), the expression of E-selectin ligands is higher in relapsed compared to newly diagnosed patients (52). In prostate cancer, the *ex vivo* interactions between circulating tumor cells (CTC) isolated from patients and E-selectin on activated endothelial cells correlated with the clinical response of castration resistant prostate cancer patients with no interactions during clinical response and many interactions at time of tumor progression (53). These data suggest a dynamic modulation of selectin ligands expression as the disease develops to a more aggressive phenotype.

Expression of selectins may also dictate to some extent tissue tropism for metastatic cancer cells. Transgenic mice overexpressing E-selectin in the liver have the propensity to develop hepatic metastasis when injected with melanoma cells expressing E-selectin ligands (54). In this system, murine melanoma cells that naturally metastasize to the lungs were engineered to express E-selectin ligands and injected in WT or transgenic mice with ubiquitously or liver specific expression of E-selectin. Liver metastases were observed only in E-selectin transgenic mice. The prevalence of liver metastasis, even in mice where E-selectin was ubiquitously expressed at similar levels in all tissues, indicates that additional factors influence the metastatic process. The large vasculature in combination with low shear stress typical of the liver may provide tumor cells with more potential adhesive sites than other organs. E-selectin dependency was also demonstrated *in vivo* for spontaneous breast metastasis to the lung and in a murine model of spontaneous liver metastasis (55, 56). In both studies, expression of E-selectin in the vasculature was dependent on pro-inflammatory and pro-angiogenic cytokines induced or even released by tumor cells, suggesting that primary tumors may promote an inflammatory activation of the endothelium in distant organs to facilitate metastatic seeding (56, 57). Selectins were also involved in engraftment of chronic myelogenous leukemia (CML) and chronic eosinophilic leukemia (CEL) in a severe combined immunodeficiency (SCID) mouse xenograft model (58). E- and P-selectin KO mice that were injected with leukemic cells displayed remarkably increased survival and little organ infiltration compared to WT mice. Moreover, the murine breakpoint cluster region-Abelson 1 positive (BRC-ABL1^+^) progenitor cells that resemble human CML demonstrated low engraftment in recipient mice deficient for E-selectin (59). Murine BRC-ABL1^+^ progenitor cells deficient either in PSGL-1, CD44, and FUT4, 7 or core2 β1-6-*N*-acetylglucosaminyl-transferase (core2 GlcNAcT-I) were impaired for engraftment.

Selectins other than E-selectin are also implicated in tumor extravasation and homing. In the previous study, L-selectin was also shown to be important for BRC-ABL1^+^ progenitor cells homing in the bone marrow (59). Recently, L-selectin has been shown to be one of the key receptors for chronic lymphocytic leukemia (CLL) homing and extravasation into the lymph nodes, an important niche where CLL cells proliferate and become resistant to chemotherapy (60). L-selectin was also shown to facilitate tumor metastases by recruiting leukocytes at sites of tumor embolization (61). Leukocytes may assist extravasation by bridging tumor cells or thrombi to the vascular endothelium and breaching the endothelial barrier. Indeed, E-selectin is frequently upregulated in the endothelium of the metastatic environment (62). Since L-selectin is an E-selectin ligand (63, 64), E-selectin present on the metastatic endothelium may contribute to leukocyte recruitment and establishment of a metastatic niche. Recently, the expression of PSGL-1 on monocytes has been shown to promote tumor extravasation (65). Although the type of selectin binding to PSGL-1 was not determined, this study further emphasizes the important role of selectins/selectin ligands interaction in tumor extravasation. P-selectin was found to be important in a metastatic model of melanoma and breast cancer (66). In this study, B16F1 melanoma cells were able to metastasize in the lungs and liver of NK-depleted/deficient mice in a P-selectin-dependent manner. Intriguingly, while metastases in the lungs were dependent on P-selectin expression on platelets and endothelial cells, hepatic metastases relied only on endothelial P-selectin. From these and other studies, it is clear that selectins and their ligands participate in the extravasation of cancer cells and they also contribute, together with other factors, to the site-specific colonization of metastatic cells.

Cancer cells exhibit a metabolic shift from oxidative to anaerobic glycolysis, this is known as the Warburg effect, which corresponds to increased gene expression of sugar transporters and glycolytic enzymes in cancer cells and is part of the adaptation of cancer cells to a hypoxic tumor microenvironment. These changes have been recently linked to induction of genes related to the expression of SLe^x^ in cancer (67). This includes ST3Gal1, FUT7, and uridine 5` diphosphate (UDP)-Gal transporter-1 (UGT1), which are induced when colon cancer cells are grown under hypoxic conditions; interestingly, this is believed to be mediated by hypoxia inducible factor (HIF) (68). This leads to higher expression of SLe^x^ and SLe^a^ on cancer cells and is likely to at least partially explain the increased SLe^x^- determinant expression seen in some cancers, which may be accompanied by a concomitant increase in E-selectin binding activity. We have made a similar observation in MM. When grown in culture under hypoxic conditions (1% O_2_), we have seen marked upregulation of ST3Gal6 transcripts along with a significant increase in E-selectin ligand expression (69). This process refers to the “neosynthesis” hypothesis related to the mechanism of enhanced expression of carbohydrate determinants of selectin ligands in cancers (70, 71). This was further examined in a study looking at the association between SLe^x^ and SLe^a^ expression on colon cancer cells and epithelial–mesenchymal transition (EMT). Induction of EMT was shown to increase SLe^x^ and SLe^a^ expression and enhance E-selectin binding. In this study, transcript levels of ST3Gal1/3/4 and FUT3 were significantly elevated and found to be regulated by c-Myc. This study outlines the role of SLe^x^ and SLe^a^ expression in mediating selectin binding during EMT (72). It is thus possible that hypoxia and other drivers of EMT may lead to the mobilization of selectin ligand-bearing cells that are then capable of homing to distant sites where selectins are expressed.

### Selectins and Tumor Emboli Formation

The first association between cancer and thrombosis, and subsequent microemboli formations, came from Trousseau in 1895, and since then, this association was further established by a large body of clinical evidence (73). Indeed, cancer patients often experience malignancy-associated thrombosis, which is the second commonest cause of cancer mortality (74). In cancer patients and in particular in metastatic cancer patients, the risk on thrombosis is associated with platelet hyperactivity (75). Cancer cells induce activation of platelets by different types of mechanisms; however, the common outcome of this activation is an increase in metastatic spread of cancer cells (73, 76). The link between platelets and cancer metastases seems to be a common feature of different types of cancers. Moreover, platelets contribute to the metastatic process in many ways including enhancing tumor cell adhesion in the vasculature, a process where P-selectin seems to have a prominent role (74, 76). Indeed, mice deficient of P-selectin had fewer metastases in the lungs than WT mice when injected with colon cancer cells (77). In the same study, it was also shown that cancer cells in the lung vasculature were surrounded by mouse platelets, an event that was greatly reduced in P-selectin^−/−^ mice. Similarly, lung metastases were significantly reduced in P-selectin^−/−^ mice in a syngeneic model of metastatic melanoma (78). However, when P-selectin was selectively deleted in platelets, melanoma metastasis were attenuated but not at the same extent as the KO mice, uncovering an essential role of endothelial P-selectin in lung metastasis. Subsequent studies highlighted that the absence of platelets not only attenuate melanoma metastasis in the lungs but also increase liver metastases, suggesting that platelets may specifically direct metastases in the lungs (66). Organ-specific vasculature anatomy, as well as hemodynamic, may explain in part the different requirement of platelets as they may assist tumor cell attachment in the lungs where the blood flow is naturally fast but are dispensable in the liver, where P-selectin expressed on endothelial cells is sufficient to capture tumor cells due to inherently slow blood stream. Formation of tumor emboli or tumor aggregates may also directly regulate the expression levels of selectin ligands on tumor cells. Cells from the metastatic breast cancer line MDA-MB-231 do not roll on E-selectin *in vitro* (79). However, when cultured in 3D spheroids, these cells upregulate E-selectin ligands and exhibit robust rolling on E-selectin *in vitro*. Induction of E-selectin ligands on spheroids may depend on the secretion of pro-inflammatory cytokines, such as interleukin 6 (IL6) and TNFα.

### Selectin and the Establishment of a Metastatic Niche

The predisposition of certain organs to support metastases of specific cancer is long recognized and represents the basis of the “seed and soil” hypothesis proposed by Steven Paget more than 100 years ago (80). The generation of a permissive microenvironment or a premetastatic niche to enable circulating cancer cells to colonize distant organs is crucial for an efficient establishment of metastases (80, 81). Several factors contribute to the formation of a premetastatic niche, such as primary tumor-derived secreting factors, exosomes, bone marrow-derived cells, and stromal cells. Selectins and their ligands may also contribute to shape the metastatic niche. In a syngeneic mouse model of lung metastases, it has been shown that vascular endothelial growth factor (VEGF) and other tumor-derived secreting factors promote focal adhesion kinase- (FAK)-dependent expression of E-selectin in the lung endothelium, thereby facilitating cancer cell homing in the lungs (56). Since selectins are one of the main mediators of leukocytes trafficking, it is conceivable that selectins contribute to leukocyte recruitment at metastatic and premetastatic sites. P- and L-selectin-mediated interactions between tumor cells, platelets, and leukocytes have been shown to induce endothelial activation with concomitant induction of C–C Chemokine Ligand 5 (CCL5) expression *in vitro* and *in vivo* (82). Moreover, CCL5 expression could be blocked by an anti-P-selectin blocking antibody demonstrating the importance of selectins in this process. Monocyte homing to metastatic sites was dependent on the expression of CLL5 from the activated endothelium, and remarkably, inhibition of monocyte recruitment greatly decreased metastases by reducing cancer cell survival. Subsequently, it was shown that monocyte recruitment at metastatic site was dependent on monocytic expression of PSGL-1, a well characterized ubiquitously selectin ligand (65). In this study, the MC-38 GFP colon carcinoma cell line showed increased numbers of metastasis in the lungs of C57BL/6 mice compared to the FUT7-deficient mice. Metastases were rescued by injecting selectin ligands bearing leukocytes, highlighting the importance of selectins/selectin ligands interactions in establishing a prometastatic niche.

### Selectin and Cancer Cells Retention in the Niche

It has been demonstrated that E-selectin regulates hematopoietic stem cells (HSC) dormancy in the bone marrow niche (83). Indeed, HSC quiescence and self-renewal were enhanced in E-selectin^−/−^ mice. Although to our knowledge, there is no evidence for such a role of selectins in solid tumors, retention of leukemic cells in their niche might be mediated in part by selectins. An example is represented by AML where E-selectin plays a role in retention and protection of the leukemic stem cells (LSC) in the bone marrow vascular niche (84). Indeed, LCS from an AML mouse model, where E-selectin was knocked out, showed greater sensitivity toward cytarabine compared to WT AML mice. Moreover, treatment with GMI-1271, a small molecule glycomimetic, rationally designed based on the bioactive conformation of SLe^a/x^ and a potent and specific antagonist of E-selectin, caused mobilization of the LCS into the blood, suggesting that E-selectin contributes to the retention of LSC in the bone marrow vasculature niche. Within the bone marrow niche, E-selectin might protect AML cells by regulating key pathways, such as Wnt [Wingless-type mouse mammary tumor virus (MMTV) integration site family member] and sonic Hedgehog (52). E-selectin was shown to regulate cell cycle in LSCs of a murine retroviral transduction/transplantation model of CML, suggesting that E-selectin may also be an important component of the bone marrow vascular niche in CML (85). In MM, PSGL-1/P-selectin interactions seem to be important not only for homing of malignant cells to the bone marrow but also in mediating the interactions between the malignant cells and the bone marrow niche, where PSGL-1 regulates myeloma proliferation and resistance to therapy (35).

## Targeting the Selectins and Their Ligands

**Figure 3:**
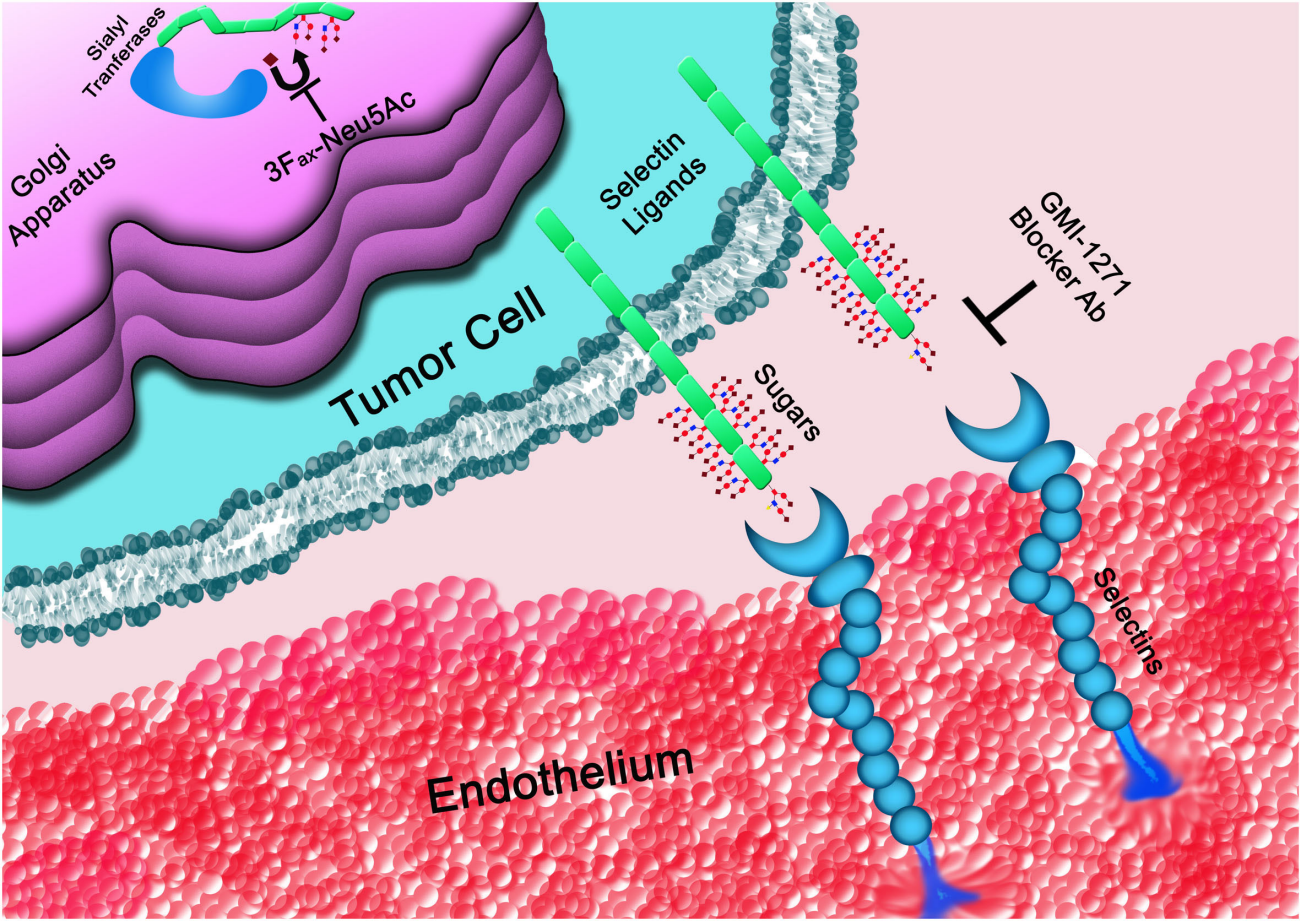
**The glycans determinants required for selectins binding are generated within the Golgi apparatus**. STs, such as ST3GAL6, are responsible for the covalent linkage of sialic acid (♦) to the underline sugar backbone. Once on the cell surface, selectin ligands interact with selectins mediating cell rolling on the vasculature endothelium. Interactions of tumor cells with platelets or the endothelium (represented in the figure) can be blocked at the level of the Golgi apparatus by inhibiting STs or by directly interfering with the binding to selectins using blocker antibodies (Ab) or small molecules, such as GMI-1271, among others.

Inhibition of selectin–selectin ligand interactions could impact different aspects of the tumor from metastatic spread to reshaping the metastatic niche. The consequence of this strategy depends certainly on the type of cancer and the stage at diagnosis. Inhibition of selectin/selectin ligand interactions can be achieved by different strategies, including inhibition of key enzymes responsible for generating the selectin ligands, such as STs, or blockage of selectin/selectin ligands interaction (Figure 3); in the latter case, the use of a variety of compounds can be employed spanning from small molecules, glycomimetics, heparin derivatives, and blocking antibodies.

### ST Inhibition

**Figure 4:**
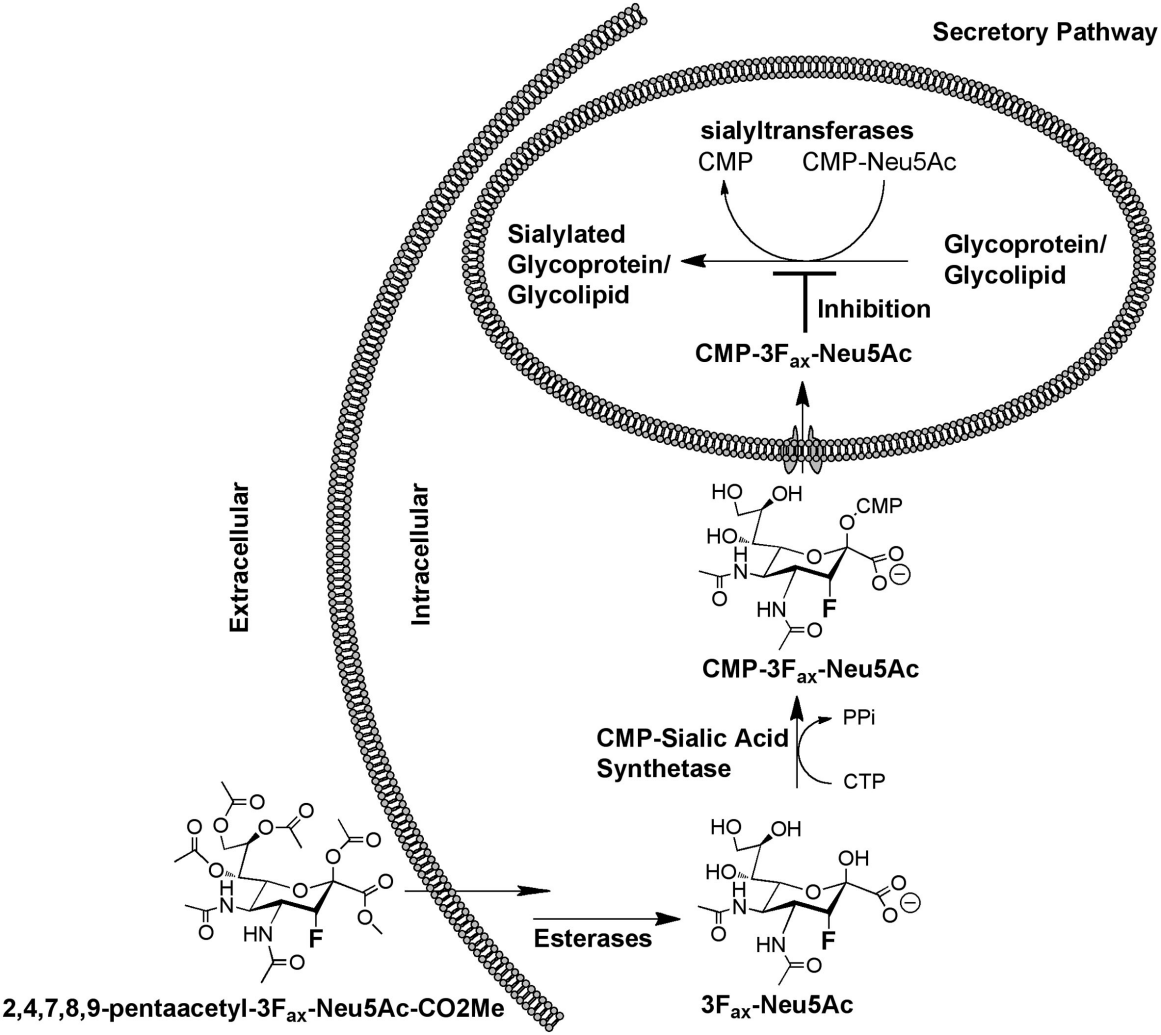
**Mechanism of action of the CMP-3F_ax_-Neu5Ac inhibitor (see text for details)**.

Due to the broad scope of biological processes regulated by sialic acid-containing glycoconjugates in both health and disease, many studies have aimed to develop ST inhibitors (86). While several of these have successfully develop potent inhibitors with partial selectivity against individual family members *in vitro*, the large majority of the developed compounds are not cell permeable, since they incorporate CMP or a derivative thereof giving the compounds negative charge and not enabling them to cross the cell membrane, let alone into the Golgi apparatus where their targets are located. A breakthrough in a general approach to developing glycosyltransferase inhibitors came when Vocadlo and colleagues introduced a Trojan Horse approach that involves the use of the cells’ own metabolic machinery to convert an unnatural monosaccharide into its corresponding nucleotide diphosphate donor substrate (87). Paulson and colleagues subsequently applied this approach to the development of the first cell permeable ST inhibitor described as 3F_ax_-Neu5Ac (88). Delivered to cells in its peracetylated form enables it to readily diffuse across the cell membrane, be deacetylated by cellular esterases and subsequently be formed into CMP-3F_ax_-Neu5Ac (Figure 4), which acts as a very poor substrate for the STs by virtue of the electron withdrawing effects of the fluorine substituent (89). As a consequence, CMP-3F_ax_-Neu5Ac builds up to high levels inside the cells and impairs the actions of all mammalian STs examined to date, both *in vitro* (88) and *in vivo* (90).

The therapeutic potential of 3F_ax_-Neu5Ac in reversing the hypersialylated state of cancer cells was realized by Adema and colleagues, where they demonstrated that treating skin melanoma with 3F_ax_-Neu5Ac prior to injecting cells into the mice greatly impaired tumor growth *in vivo* (91). These results nicely demonstrated the potential of ST inhibition as a cancer therapeutic. Nevertheless, due to critical roles played by sialylation in healthy tissues, the non-selective nature of this inhibitor is a concern. In particular, an important role for sialylation was confirmed within the kidney in a study where 3F_ax_-Neu5Ac was systemically administered to mice (90). At certain doses, 3F_ax_-Neu5Ac caused irreversible kidney damage, resulting in loss of protein in within the urine, significant loss of weight, and the eventual death of the animals. A similar phenotype has also been observed in mice deficient in sialic acid biosynthesis (92). It is noteworthy that out of the numerous ST-deficient mice established to date, none have developed such a phenotype. Based on these observations, it is likely that multiple STs work together to form the negatively charged glomerular filtration system on podocytes within the kidney. Hence, selectively inhibiting an individual ST *in vivo* may be feasible without the severe secondary consequences to the host observed with the global ST inhibitor.

A more complete structural knowledge of the ST family may enable selective ST inhibitors to be developed. Without these in hand, however, Adema and colleagues have developed an innovative approach to selectively deliver 3F_ax_-Neu5Ac to cells *in vivo* (22). Working with poly lactic acid (PLGA) nanoparticles to encapsulate 3F_ax_-Neu5Ac, the compound was successfully delivered to melanoma cells by virtue of an antibody displayed on the surface of the nanoparticles that is specific for a tumor antigen. Targeting 3F_ax_-Neu5Ac to the cancer cells in this way allowed for delivery of the compound to cells within mice, preventing metastasis of the melanoma cells to the lung. This approach appears to have broad applicability for different forms of cancer, so long as a specific tumor antigen is known. While work in this area is still in its infancy, modulating ST activity with small molecule inhibitors is an exciting avenue with broad therapeutic potential.

### Small Molecules, Blocking Antibodies, Glycomimetics, and Heparin Derivatives

Inhibition of P-selectin has been shown to reduce metastasis *in vivo* in different types of tumor. In MM, PSGL-1 has been shown to regulate the interaction between the malignant cells and their microenvironment, including macrophages (35, 93). Moreover, PSGL-1/P-selectin interactions mediate homing of the myeloma cells to the bone marrow and resistance to chemotherapy in the context of the bone marrow niche *in vivo*. Importantly, inhibition of PSGL-1/P-selectin interactions by the glycomimetic compound GMI-1070, or by humanized blocker antibodies to P-selectin or PSGL-1, was able to suppress homing of myeloma cells to the bone marrow and restore sensitivity to bortezomib (35, 50). Recently, holothurian glycosaminoglycan (hGAG), a marine compound similar to heparin sodium, has been shown to decrease melanoma lung metastasis in mice possible through the inhibition of P-selectin although other mechanisms may account for this effect (94). Metastases are attenuated by heparin and low-molecular fraction heparins whose mechanism(s) of action, among others, is the suppression of platelets–tumor cells and endothelial–tumor cells interactions following P-selectin inhibition (95–97). Similar mechanism(s) is shared by other glycan mimetics or molecules, such as sulfated hexasaccharides and ascidian dermatan sulfates (98, 99).

Although MM universally express ligands for P-selectin, we have recently shown that rolling on E-selectin is restricted to a minority of myeloma cells expressing functional E-selectin ligands (51, 69). Moreover, when xenotransplanted into a SCID mouse model, the E-selectin ligands expressing myeloma cells gave rise to a remarkably aggressive disease characterized by resistance to bortezomib (51). Notably, the glycomimetic E-selectin inhibitor GMI-1271 improved survival and restored the anti-myeloma activity of bortezomib. GMI-1271 was also able to reduce leukemia burden in primary AML blasts engrafted non-obese diabetic (NOD) SCID IL2 receptor γ(c) null (Rγc^−/−^) mice when combined with chemotherapy (52). GMI-1271 in combination with standard chemotherapy showed promising results in reducing metastatic lesions in xenograft mouse models of pancreatic and breast cancer (100, 101). In the latter, it was also shown that GMI-1271 targets MCF7 breast cancer stem cells (CD44^+^CD24^−^) homing to the bone marrow, pointing out a role of E-selectin in bone marrow dissemination of cancer stem cells in breast cancer (102). A first *in human* experience with GMI-1271 demonstrated favorable safety together with no mobilization of human stem cells (103). Targeting E-selectin may also be beneficial in alleviating the side effects of chemotherapy and, therefore, improve patient survival and quality of life, especially in elderly and vulnerable patients. In this respect, inhibition of E-selectin by GMI-1271 was shown to alleviate mucositis, resulting from the recruitment of inflammatory macrophages to the damaged intestine after chemotherapy. Indeed, a marked upregulation of E-selectin was detected in the intestine following chemo or radiotherapy, which is critical to the recruitment of inflammatory cells to the mucosa (104).

Based on these results and the promising preclinical findings in AML, a clinical trial to determine the safety, pharmacokinetics, and efficacy of GMI-1271 in combination with chemotherapy in AML is currently going on (NCT02306291). Moreover, based on the emerging data implicating E-selectin and its ligands in MM, a phase I trial evaluating GMI-1271, as an adjunct to bortezomib-based therapy, is planned. Molecules similar to GMI-1271 have also been developed, in particular GMI-1359, which exhibits dual specificity toward E-selectin and CXCR4. In combination with standard chemotherapy, this molecule showed the ability to reduce metastases in xenograft mouse models of pancreatic and prostate cancers and improved survival in a xenograft mouse model of AML (105–107).

### Exploiting Selectin/Selectin Ligand Interactions

Targeting selectins may also represent an attractive strategy to deliver chemotherapeutic drugs to sites of metastases. Melanoma lung metastases were decreased by an E-selectin-targeted polymer–drug conjugates. In this study, an E-selectin binding peptide was conjugated with *N*-(2-hydroxylpropyl) methacrylamide and equipped with doxorubicin or a proapoptotic peptide that can be released at mild pH conditions (108). The copolymer effectively decreased melanoma lungs metastases and significantly improved mice survival. As melanoma cancer cells induce the expression of E-selectin in the lung and liver, the copolymers were accumulation primarily in these tissues. A drug-free copolymer was also able to prevent lung metastases, suggesting decreased cancer cells extravasation due to inhibition of E-selectin/selectin ligands interactions. Similarly, low-molecular-weight, heparin-coated, doxorubicin-loaded liposomes were shown to inhibit melanoma metastases to the lungs by inhibiting tumor cells platelets formations (109).

Leukocytes have been functionalized by selectin-coated liposomes to deliver proapoptotic molecules, such as tumor necrosis factor related apoptosis inducing ligand (TRAIL), to tumor cells. E-selectin-coated liposomes containing TRAIL were used to “equip” circulation leukocytes with TRAIL by taking advantage of the interactions between E-selectin and its ligands expressed on the surface of leukocytes (110, 111). The “equipped” leukocytes were able to efficiently clear circulating colon cancer and prostate cancer cells in a xenograft mouse model.

## Future Perspectives

In conclusion, there is mounting evidence supporting a role for selectins and their ligands in cancer patients, promoting metastatic behavior and disease progression. The emergence of new therapeutic strategies will hopefully lead to an improvement in outcome of patients with aberrant selectin ligand expression.

## Author Contributions

MO contributed to writing the manuscript, communicated with journal editor, and submitted the manuscript. AN and MM contributed to writing the manuscript and preparing the figures.

## Conflict of Interest Statement

MO has received research support from GlycoMimetics Inc. AN and MM have no conflict of interest to declare.
